# Production of Polyclonal Antibody to the HPV58 E7 Protein and Its Detection in Cervical Cancer

**DOI:** 10.1371/journal.pone.0169138

**Published:** 2016-12-29

**Authors:** Qiaoli Zheng, Tuan Wang, Shaojie Jiang, Rui Han, Na Jin, Jiang Zhu, Qiang Zhou, Hui Wang, Xianzhen Chen, Hao Cheng

**Affiliations:** 1 Department of Dermatology, Sir Run Run Shaw Hospital, School of Medicine, Zhejiang University, Hangzhou, Zhejiang Province, China; 2 Department of Radiology, Sir Run Run Shaw Hospital, School of Medicine, Zhejiang University, Hangzhou, Zhejiang Province, China; 3 Department of Pathology, The First People’s Hospital of Fuyang Hangzhou, Hangzhou, Zhejiang Province, China; Penn State University School of Medicine, UNITED STATES

## Abstract

The persistent infection of high-risk human papillomavirus (HPV) is one of the most common causes of cervical cancer worldwide, and HPV type 58 is the third most common HPV type in eastern Asia. The E7 oncoprotein is constitutively expressed in HPV58-associated cervical cancer cells and plays a key role during tumorigenesis. To study the biological function of HPV58 E7 and to characterize E7 protein-host cell interactions, we cloned the human HPV58 E7 gene and produced specific E7 antibodies. The HPV58 E7 gene was cloned into a prokaryotic expression vector, pGEX-4T2. The recombinant plasmid pGEX-4T2-(HPV58-E7) was transformed into *Escherichia coli* DH5α and expressed as a fusion protein containing a GST tag. After purification and removal of the GST affinity tag, the E7 protein was used as an antigen for the production of antiserum in rabbits. The specificity of the purified HPV58 E7 antibody was detected by western blotting, immunofluorescence and immunohistochemistry analysis. These methods demonstrated that the polyclonal antibody could specifically recognize the endogenous and the recombinant HPV58 E7 proteins. Immunohistochemistry analysis indicated that the E7 protein was localized in the nucleus of cervical cancer cells.

## Introduction

Cervical cancer is one of the most common female genital cancers, with an estimated 53,000 new cases and 28,000 deaths occurring each year around the world [1]. The high-risk human papillomavirus (HPV) is a primary cause of cervical cancer [2]. HPV58, a common subtype of high-risk HPV, plays a more prominent role in HPV-associated cervical cancer in Asian countries. HPV58 has been found in 11.5% to 28% of cervical cancer patients in China [3].

Once HPV infection occurs, the viral genome is integrated into the host cell DNA, the biological synthesis and assembly of viral components is carried out, and parts of viruse proteins are finally released from within the host cell [4]. The high-risk HPV E6 and E7 gene-encoded oncoproteins degrade and inactivate tumor suppressor proteins, such as p53 and retinoblastoma protein (pRB), and promote the malignant transformation of the host cells [5]. Notably, a high expression level of E6 and E7 oncoproteins are biological hallmarks of HPV-associated cancers [6].

To date, a commercial antibody to HPV58 E6 and E7 are still unavailable. In this study, we aimed to express the HPV58 E7 protein *in vitro*, and further produce and verify the specific polyclonal antibody against the E7 protein. The expression of the HPV58 E7 protein and its antibody will help to investigate the function of this oncoprotein and further evaluate its clinical significance.

## Materials and Methods

### Patients and animals

This study was performed according to the guidelines set forth by the Declaration of Helsinki. The study protocol was approved by the Ethics Committee of Sir Run Run Shaw Hospital of Zhejiang University School of Medicine. Written informed consent was obtained from the participants in accordance with the requirements of the Ethics Committee of Sir Run Run Shaw Hospital of Zhejiang University School of Medicine. All the patients who participated in the study were assessed between March 2013 and March 2015. Paraffin-embedded specimens from cervical cancer patients diagnosed according to the WHO classification of tumors of female genital organs by the Division of Pathology were collected by the Departments of Obstetrics and Gynecology, Sir Run Run Shaw Hospital, School of Medicine, Zhejiang University, China. HPV 16-, 18- and 58-positive cervical epithelium cells were obtained from the Departments of Obstetrics and Gynecology, Sir Run Run Shaw Hospital, School of Medicine, Zhejiang University, China. Authors could access information that could identify individual participants during and after data collection.

Animal studies were carried out in strict accordance with the guidelines of the regional Animal Ethics Committee and the rules for experimental animals at Zhejiang University. New Zealand White rabbits (3-months old, approximately 2.5 kg) were used in the polyclonal antibody preparation. All the animals were from the same line and reared in similar conditions at the Breeding Station of Zhejiang Chinese Medical University and were sacrificed according to the standards of the Animal Management of Zhejiang University.

### Construction of HPV58 E7 prokaryotic expression vector

In situ polymerase chain reaction (PCR) was used to clone the HPV58 E7 gene from human HPV58-positive cervical epithelial cells of a patient. The specific primer pair was designed to amplify the target HPV58 E7 gene (the forward primer: 5’-CCGGGATCCATGAGAGGAAACAACCCAACG-3’, and the reverse primer: 5’-CCGGAATTCTTATTGCTGTGCACAGCTAGG-3’) and synthesized by Qingke Biological Technology Co., Ltd., Hangzhou, China. Briefly, 5 μl human HPV58-positive cervical epithelial cell solution was used for in situ PCR in a reaction (50 μl) containing 0.4 μM primers, 0.2 mM dNTP mixture (Takara, Japan), and 0.5 μl rTaq polymerase (Takara, Japan). The PCR amplification was performed on an Applied Biosystems S1000 Thermal Cycle and the products were separated on a 1.5% agarose gel. The DNA fragments were recovered from the agarose gel using the Gel Extraction kit (OMEGA Bio-Tek, U.S.A.)

The full length coding sequence of the HPV58 E7 gene was inserted into the pGEX-4T2 vector in-frame with the GST open reading frame. The HPV58-E7 gene and pGEX-4T2 vector were cleaved by *Eco*R I (Takara) and *Bam*H I (Takara), and linked by T4 DNA ligase (Takara). The reconstructed pGEX-4T2-(HPV58-E7) vector was transformed into competent *Escherichia coli* DH5α (Takara). Bacterial cells were collected from an overnight culture in LB medium by centrifugation, washed twice with PBS, and lysed for plasmid isolation using the manufacturer’s protocol (OMEGA Bio-Tek). The vector was cleaved with the restricted endonucleases *Eco*R I/*Bam*H I, followed by agarose gel electrophoresis. Positive samples were sent to Qingke Biological Technology Co., Ltd. for sequencing.

### Construction of HPV58 E7 eukaryotic expression cassette

The HPV58 E7 gene was further amplified through PCR with specific primers (the forward primer: 5’-CCGGAATTCATGAGAGGAAACAACCCAACGC-3’, and the reverse primer: 5’- CCGGGATCCTTATTGCTGTGCACAGCTAGG-3’) using the pGEX-4T2-(HPV58-E7) vector as a template DNA. The 303 bp HPV58-E7 fragment containing restriction sites for unique enzymes including *Bam*H I and *Eco*R I was inserted into the pEGFP-C1 vector with the EGFP open reading frame. The recombinant plasmid was isolated from *E*. *coli* clones and verified through restriction analysis and sequencing by Qingke Biological Technology Co., Ltd.

The vectors were transiently transfected into HEK293T cells according to the manufacturer’s instructions (Lipofectamine™ 3000, Invitrogen, U.S.A.) Briefly, HEK293T cells were cultured in DMEM with high glucose supplemented with 10% fetal bovine serum (FBS; Sijiqing, China) in a humidified 37°C incubator for transfection. The diluted pEGFP-C1-(HPV58-E7) or pEGFP-C1 vectors were added to the diluted Lipofectamine™ 3000 reagent in Opti-MEM™ medium (1:1 ratio), and incubated for 10 to 15 minutes at room temperature. Next, we added the DNA-lipid complex to the cells and analyzed the transfected cells after 2 to 4 days at 37°C.

### Expression and purification of HPV58 E7 protein

The verified pGEX-4T2-(HPV58-E7) vectors were transformed into *E*. *coli* DH5α and induced by 0.2 mM isopropyl β-D thiogalactopyranoside (IPTG; Beyotime, China), which led to the production of soluble GST-HPV58-E7 fusion protein. In brief, colonies were inoculated into LB medium (100 μg/ml Amp; Beyotime) and incubated in a high speed shaker (250 rpm) at 37°C until the OD600 nm reached 0.6–0.8 (8 h). IPTG with the final concentration of 0.2 mM was added to the medium and incubated at 26°C for 4 to 6 h. The broth medium was centrifuged at 2000 ×*g* (5 min) and the bacterial pellet was resuspended in PBS for sonication on ice. After centrifugation (2000 ×*g* for 5 min), the supernatant and the sediment were both reduced in lysis buffer and subjected to 10% SDS-PAGE. After electrophoresis of the proteins, the gel was stained in 50 ml Coomassie blue stain (0.25% Coomassie Brilliant Blue R-250, 10% glacial acetic acid, 25% methanol) for 1 h at room temperature with gentle agitation, followed by destaining in distilled water for more than 2 h until the protein bands were visible clearly.

Glutathione-Sepharose 4B beads (Life, U.S.A.) were added to the remaining supernatant for binding of the GST-HPV58-E7 protein. The purified HPV58 E7 protein was obtained after removal of GST by incubation with thrombin (Life) and dialysis in PBS overnight, and stained in Coomassie blue followed by electrophoresis on a 10% SDS-PAGE gel.

### Preparation and purification of polyclonal antibody against HPV58 E7 protein

The purified HPV58-E7 protein was mixed with an equal volume of Freund’s complete adjuvant (Sigma-Aldrich, U.S.A.) to a final concentration of 300 μg/ml. Subsequently, approximately 500 μg of HPV58-E7 protein was injected subcutaneously into the backs of New Zealand White rabbits (3-months old, approximately 2.5 kg). The following three times, the immunizations were executed with HPV58-E7 protein in Freund’s incomplete adjuvant at 10-day intervals. An enzyme-linked immunosorbent assay (ELISA) was used to test rabbit antisera for the presence of antibodies to HPV58-E7 and was repeated daily until a threshold was observed (1:100,000). The HPV58 E7 protein was diluted to 50 μg/ml with a 0.05 M, pH 9.6 carbonate buffer solution, and 100 μl diluted protein was added to each polystyrene board at 4℃ overnight. Then, the board was washed with PBST (PBS plus 0.05% Tween 20) three times and blocked with 200 μl 5% milk at 37℃ for 2 h. After washing, the HPV58 E7 antibody was diluted 1:100,000 times, and 100 μl was added to the board at 37℃ for 1 h. The secondary HRP-conjugated goat anti-rabbit antibody was added, followed by incubation with TMB solution and termination with 2 M sulfuric acid. The measurement of the color produced was quantified with a spectrophotometer at 450 nm wavelength, the OD of the positive sample was 2.1 times that of the negative sample. After that, another 500 μg HPV58-E7 protein without adjuvant was injected intramuscularly into the rabbits’ legs 10 days later. Then, 5 days after the last injection, blood was collected through the carotid artery of the rabbits under anesthesia. We collected 100 to 120 ml blood from each rabbit until the rabbit died. The serum was separated and preserved in -20°C.

The rProtein G Agarose (Life) was applied for the isolation of IgG antibodies using column chromatography according to the manufacturer’s protocol. In brief, an empty column (5 ml) was prepared with a 1 ml bed volume by loading 2 ml of 50% rProtein G Agarose slurry. Next, 10 ml of binding buffer was added to equilibrate the column before loading the rabbit serum. Subsequently, 6 to 12 ml binding buffer was added to remove unbound protein following the application of 6 ml elution buffer to elute the bound IgG antibodies.

### Western blotting analysis

Cell lysates or protein samples prepared in reducing sample buffer (50 mM Tris-HCl (pH 6.8), 2% SDS, 0.01% Bromophenol Blue, 10% glycerol, 50 mM DTT) were subjected to SDS-PAGE and transferred to polyvinylidene fluoride (0.2 μm PVDF, Bio-Rad, U.S.A.) membranes. After blocking with 10% nonfat milk (BD Difco^TM^, U.S.A.) for 1 hour, the membranes were incubated with antibodies specific for HPV58-E7 (1:10,000). Secondary peroxidase-conjugated goat anti-rabbit IgG (1:5000; Beyotime) was incubated for 2 hours at room temperature and ECL substrates (Millipore, U.S.A.) were applied and signals were detected by a chemiluminescence imaging system (FUJIFILM, U.S.A.).

### Immunofluorescent stain

The 293T-pEGFP-C1 or 293T-pGEX-C1-HPV58-E7 cells were seeded in a 6-well plate with glass-bottomed dish for 1 day. The cells were washed, fixed with 4% paraformaldehyde (Bio-Rad) for 10 min, and permeabilized with 0.1% Triton X-100 (Solarbio, China) for 10 min. After washing with PBS three times, the cells were blocked with 5% goat serum (Beyotime) and incubated with primary antibodies (anti-HPV58-E7; 1:400) overnight at 4°C. The cells were washed and further incubated with secondary antibodies (Alexa Fluor 555-conjugated donkey anti-rabbit IgG; 1:500; Beyotime). The nuclei were stained with 200 μg/ml DAPI (Beyotime). The stained cells were observed under a microscope (OLYMPUS, U.S.A.).

### Immunohistochemistry analysis

Formalin-fixed paraffin-embedded (FFPE) HPV58-positive (detected by PCR) endometrial cancer sections (8-μm) were used for immunohistochemical staining of the HPV58-E7 protein. Endogenous peroxidases in the de-paraffinized sections were quenched with 0.3% hydrogen peroxide in 60% methanol for 20 min. Non-specific adsorption was minimized by incubating the sections in 2% normal goat serum in PBS for 20 min. The sections were then incubated with anti-HPV58-E7 rabbit polyclonal antibody (1:500) at room temperature for 2 hours. Then, the sections were washed with PBS and incubated with an HRP-conjugated goat anti-rabbit IgG secondary antibody (1:1000). The immunoreactions were visualized by incubating the sections for 3 minutes in a 0.1% 3,3′-diaminobenzidine (DAB) solution and counterstained with hematoxylin for 8 mins. The stained cells were observed under a microscope (OLYMPUS, U.S.A.).

## Results

### Construction of expression vector of the HPV58 E7 gene

**Fig 1:**
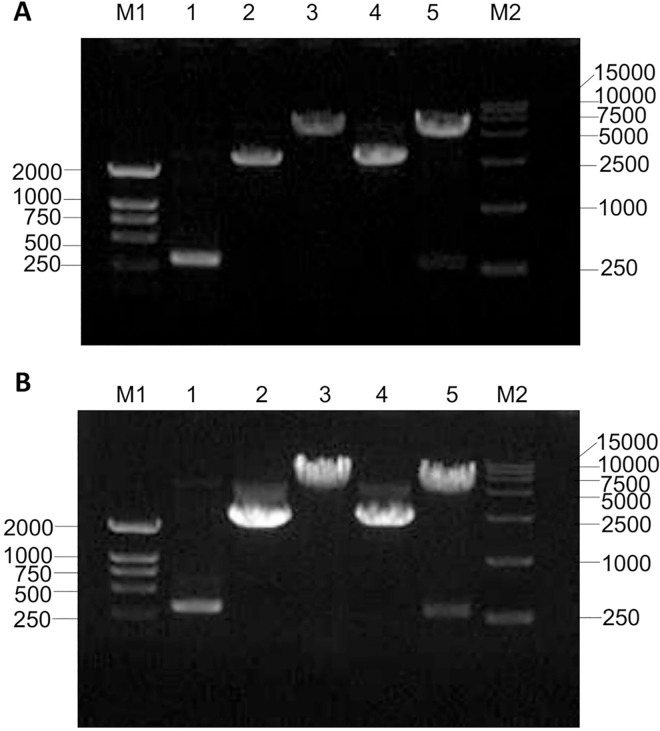
Construction of the expression vector of the HPV58 E7. (A) Construction of pGEX-4T2-(HPV58-E7) vector. The lane M1 shows the TaKaRa DL2000 DNA marker, lane 1 shows the HPV58 E7 gene (PCR from human HPV58-positive cervical epithelial cells); lane 2 is the pGEX-4T2 vector; lane 3 is the cleaved pGEX-4T2 vector by *Eco*R I and *Bam*H I; lane 4 shows the reconstructed pGEX-4T2-(HPV58-E7) vector; lane 5 is the verification of pGEX-4T2-(HPV58-E7) vector which was cleaved by *Eco*R I and *Bam*H I; the lane M2 is TaKaRa DL 15,000 DNA marker. (B) Construction of pEGFP-C1-(HPV58-E7) vector. The lane M1 shows the TaKaRa DL2000 DNA marker, lane 1 shows the HPV58 E7 gene (PCR from pGEX-4T2-(HPV-58E7) vector); lane 2 is the pEGFP-C1 vector; lane 3 is the cleaved pEGFP-C1 vector by *Eco*R I and *Bam*H I; lane 4 shows the reconstructed pEGFP-C1-(HPV58-E7) vector; lane 5 is the verification of pEGFP-C1-(HPV58-E7) vector which was cleaved by *Eco*R I and *Bam*H I; the lane M2 is TaKaRa DL 15,000 DNA marker.

The present study aimed to express HPV58 E7 protein *in vitro* and produce its antibody. To this end, the HPV58 E7 gene was first inserted into the pGEX-4T2 and pEGFP-C1 vectors between the restriction sites for *Eco*R I and *Bam*H I (Fig 1). The recombinant plasmids pGEX-4T2-(HPV58-E7) (Fig 1A) and pEGFP-C1-(HPV58-E7) (Fig 1B) were identified by double-enzyme digestion with *Eco*R I and *Bam*H I. The sequencing further confirmed that the HPV58 E7 gene was in accordance with the design and was 297 bp in length (S1 File). The constructed expression vector of pGEX-4T2-(HPV58-E7) was introduced into *E*. *coli* DH5α, which resulted in the expression of the GST-HPV58-E7 fusion protein. The pEGFP-C1-(HPV58-E7) vector was transfected into HEK293T cells and further used in the verification of the HPV58 E7 antibodies.

### Expression and purification of HPV58 E7 protein

**Fig 2:**
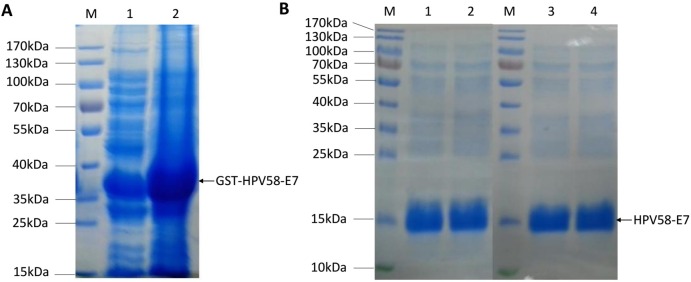
Expression and purification of HPV58 E7 protein. **(A)** Coomassie blue staining for recombinant GST-HPV58-E7 protein. Lane M is the protein ladder; Lane 2 shows the protein of the supernatant of the bacterial lysate after centrifugation; Lane 3 shows the protein of the sediment of bacteria lysate. The arrowhead indicates GST-HPV58-E7 protein. (B) Coomassie blue staining for purified HPV58-E7 protein. Lane M is the protein ladder; Lane 1 and 2 show the purified E7 protein after removal of GST-tag; Lane 3 and 4 show the E7 protein after dialysis in PBS. The arrowhead indicates HPV58-E7 protein.

The *E*. *coli* strain DH5α was transformed with recombinant pGEX-4T2-(HPV58-E7) vector, and the HPV58 E7 protein as a fusion protein with a GST-tag was expressed. After sonication and centrifugation, the proteins were separated by SDS-PAGE and visualized by Coomassie blue staining (Fig 2A). The fusion protein was further purified using Glutathione-Sepharose 4B beads and the GST-tag was removed by thrombin. SDS-PAGE revealed that the apparent molecular weight of the HPV58 E7 protein was approximately 15 kDa (Fig 2B). The results showed that the eluted protein exhibited a purity of above 95%.

### Verification of polyclonal antibody against HPV58 E7

**Fig 3:**
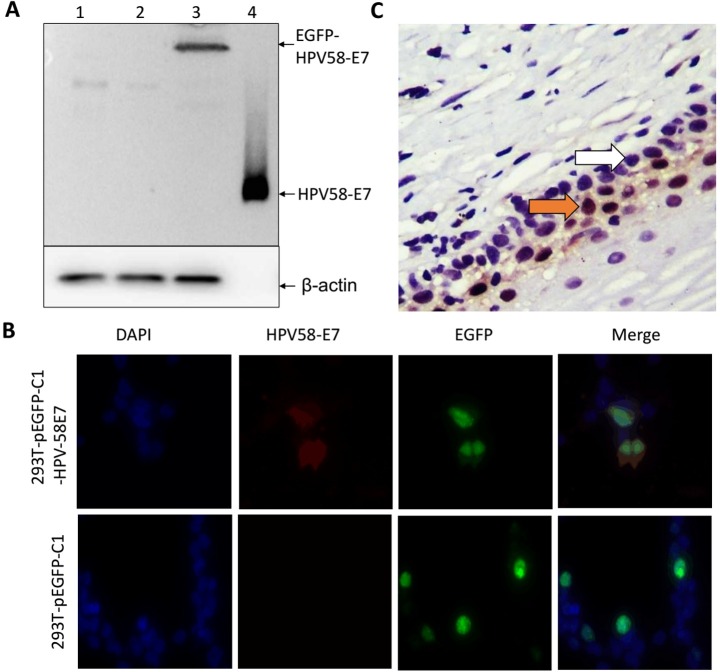
Verification of polyclonal antibody against HPV58 E7. (A) western blotting for the detection of HPV58 E7. Lanes 1, 2 and 3 are the cell lysates of HEK293T, 293T-pEGFP-C1, and 293T-pEGFP-C1-HPV58-E7, respectively; Lane 4 is the purified HPV58-E7 protein. (B) Immunofluorescent analysis for the detection of HPV58 E7 in 293T-pEGFP-C1, 293T-pEGFP-C1-HPV58-E7 cells. (C) Immunohistochemistry staining for the detection of HPV58 E7 in cervical cancer cells. Red arrowheads indicate the HPV58-positive cells. White arrowheads indicate the HPV58-negative cells.

After the rabbits were immunized five times with purified HPV58 E7 protein, the polyclonal antibody against E7 was purified using the rProtein G Agarose. The purified antibody exhibited high specificity, which was analyzed by several methods including western blotting, immunofluorescent staining and immunohistochemistry (Fig 3). Different samples were separated by 10% SDS-PAGE and transferred to PVDF membranes (Fig 3A). The predicted bands representing HPV58 E7 were found in the purified HPV58 E7 protein and total protein samples from 293T cells transfected with pEGFP-C1-(HPV58-E7) vectors, while proteins from the 293T cells and 293T-pEGFP-C1 cells did not generate any bands. These results indicated that the polyclonal antibody could recognize both the endogenous and the recombinant HPV58 E7 protein, which meant that the polyclonal antibody had high specificity.

As shown in Fig 3B, 293T-pEGFP-C1-HPV58-E7 cells could be detected with the HPV58 E7 antibodies we produced by immunofluorescent staining. The 293T-pEGFP-C1 cells showed no signal of HPV58 E7. The HPV58-positive endometrial cancer tissues were used as samples to test the distribution of the HPV58 E7 protein using the anti-HPV58-E7 antibody. The results of immunohistochemistry showed that the HPV58 E7 antibody could test the cells infected by HPV58, and the E7 protein was localized in the nucleus of cervical cancer cells (Fig 3C). Thus, the polyclonal antibody was able to recognize endogenous HPV58-E7 in tissues and may be a useful tool to study the function and mechanism of HPV58-E7.

**Fig 4:**
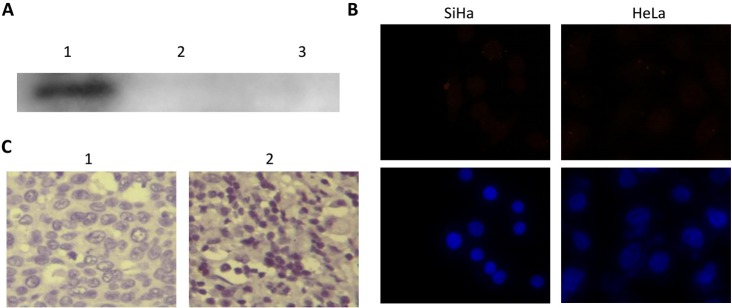
Examination of the cross-reaction of HPV 58 E7 antibody. (A) western blotting for the detection of HPV58 E7. Lanes 1, 2 and 3 are the pure protein of HPV 58, 16 and 18 E7. (B) Immunofluorescent analysis for the detection of HPV58 E7 in SiHa and HeLa cells. (C) Immunohistochemistry stain for the detection of HPV58 E7 in cervical cancer cells, which was HPV 16- or 18-positive, HPV 58 was negative. 1 is the HPV16-positive sample and 2 is the HPV18-positive sample.

Furthermore, we tested the cross-reaction between different HPV types of E7 protein antibodies through western blotting, immunofluorescent analysis and immunohistochemistry staining (Fig 4). SiHa cells are HPV 16-positive and HeLa cells are HPV 18-positive. The results showed the HPV 58 E7 antibody could not recognize the HPV 16 and 18 E7 proteins through western blotting and immunofluorescent staining in SiHa or HeLa cells (Fig 4A and 4B). We chose clinical cervical cancer samples, which were tested by PCR for HPV 16- or 18-positivity. Still, the HPV58 E7 antibody could not recognize the E7 proteins in the samples (Fig 4C).

## Discussion

Our study showed for the first time the production and application of a specific HPV58 E7 antibody. We identified the E7 protein expression both in transfectants and in clinical samples and confirmed the nuclear location of HPV58 E7 in a cervical cancer sample. Our study provides a basis for the further exploration of the HPV58 E7 protein as a potential tool for the diagnosis and detection of aggressive cervical tumors.

HPV infection is associated with a broad spectrum of mucocutaneous diseases, including condyloma acuminate and endometrial cancer. The high-risk HPV types are commonly associated with lesions that can progress into cervical carcinoma [7]. The existing studies have showed that the high-risk HPV E6 and E7 genes are the main oncogenes of high-risk HPVs [8]. The degradation of tumor suppressors p53 and pRb induced by E6 and E7, respectively, induces cellular transformation [8]. Additionally, the E7 protein would bind to a great variety of other cellular key proteins, such as p107, cyclin A and p130 [9–11]. Certain of these proteins play important roles in the induction of mitogenic pathways, in G1/S progression, and in the inhibition of cellular differentiation and may increase chromosomal instability [9–11].

Additionally, the E7 oncoprotein affected the phenotype and function of antigen-presenting cell (APCs) subsets in the skin. The E7 transgene expression in keratinocytes attracted new APC subsets to the epidermis measured by distinct cell surface markers. However, the antigen-processing was significantly impaired, which was measured by proteolytic cleavage of DQ-OVA and the activation of T cells by APCs [12]. Furthermore, there was a study that found that E7 mRNA expression was higher in women with high grade squamous intraepithelial lesion (HSIL) and cervical intraepithelial neoplasia (CIN) grade 2 or higher [13]. HPV E7 may also induce the upregulation of RRM2, which then promotes cervical carcinogenesis via ROS-ERK1/2-HIF-1α-VEGF-induced angiogenesis [14].

HPV-58, a common subtype of high-risk HPV, accounts for a substantial proportion of the cervical cancers in Asian countries. It has been found in 11.5 to 28% of cervical cancer patients in China, 16% in Korea and 8% in Japan [15]. In a recent study that evaluated the prevalence of high-risk HPVs in cervical intraepithelial lesions in Shanghai showed that the three most dominant HPV genotypes were HPV16, 58, and 52 [16]. HPV58 is the third most common HPV type in Eastern Asia overall [17]. Therefore, studying the biological function of HPV58, especially the E7 oncoprotein, would provide novel insights into HPV-associated cervical cancer development, especially following the widespread administration of HPV16/18 vaccines. Up to date, research studying the HPV58 type has been limited, and may be due to a lack of specific proteins and antibodies. Furthermore, antibodies raised against specific HPV type antigens often do not cross-react with other types, which may also be an obstacle for further research.

In the present study, we successfully expressed the HPV58 E7 protein *in vitro* and applied it in the preparation of a specific polyclonal antibody against the E7 protein. Furthermore, we verified the specificity of HPV58 E7 antibody through western blotting, immunofluorescence and immunohistochemistry analysis. The E7 protein may be used as an adjuvant for HPV58 vaccine development. The antibody can be extensively used in both biological research and clinical detection, including the western blotting, immunofluorescence, immunohistochemistry, ELISA development and flow cytometry.

In conclusion, the expression of HPV E7 protein may provide a basis for the production of HPV58 vaccine. The development of the E7 antibody will provide a new and reliable method to detect HPV58 infection in the clinical setting. These findings provide novel insight into the possible functions of the HPV58 E7 protein and serve as an important first step for the further investigation of the roles of HPV58 E7 in cervical cancer development.

## Supporting Information

S1 File The sequencing of pGEX-4T2-(HPV58-E7) and pEGFP-C1-(HPV58-E7) vector. (RAR)

S1 Fig Detection of polyclonal antibody against HPV16 and 18 E7. (A) western blotting for the detection of HPV16 and 18 E7. The upper band was marked with HPV18 E7 antibody. The lower band was marked with HPV16 E7. The Lanes 1, 2 and 3 are the pure protein of HPV 58, 16 and 18 E7. (B) Immunofluorescent analysis for the detection of HPV16 E7 in SiHa cells and HPV18 E7 in HeLa cells. (C) Immunohistochemistry stain for the detection of HPV16 and 18 E7 in cervical cancer cells. 1 is the HPV16-positive sample and 2 is the HPV18-positive sample. Red arrowheads indicate the positive cells. White arrowheads indicate the negative cells.
(TIF)

## References

[1] Jemal A, Bray F, Center MM, Ferlay J, Ward E, Forman D. Global cancer statistics. CA Cancer J Clin. 2011;61(2):69–90. 10.3322/caac.20107 21296855

[2] Yang HJ. Aberrant DNA methylation in cervical carcinogenesis. Chin J Cancer. 2013;32(1):42–48. 10.5732/cjc.012.10033 22943599 PMC3845585

[3] Ding T, Wang X, Ye F, Cheng X, Ma D, Lu W, et al Distribution of human papillomavirus 58 and 52 E6/E7 variants in cervical neoplasia in Chinese women. Gynecol Oncol. 2010;119(3):436–443. 10.1016/j.ygyno.2010.08.032 20870281

[4] Li J, Wang X, Liu J, Wang H, Zhang XL, Tang W, et al Replication and transcription of human papillomavirus type 58 genome in Saccharomyces cerevisiae. Virol J. 2010;7:368 10.1186/1743-422X-7-368 21156081 PMC3016283

[5] Howie HL, Katzenellenbogen RA, Galloway DA. Papillomavirus E6 proteins. Virology. 2009;384(2):324–334. 10.1016/j.virol.2008.11.017 19081593 PMC2674106

[6] Ghittoni R, Accardi R, Chiocca S, Tommasino M. Role of human papillomaviruses in carcinogenesis. Ecancermedicalscience. 2015;9:526 10.3332/ecancer.2015.526 25987895 PMC4431404

[7] zur Hausen H. Papillomaviruses and cancer: from basic studies to clinical application. Nat Rev Cancer. 2002;2(5):342–350. 10.1038/nrc798 12044010

[8] Fan X, Chen JJ. Regulation of cell cycle progression and apoptosis by the papillomavirus E6 oncogene. Crit Rev Eukaryot Gene Expr. 2004;14(3):183–202. 15248815 10.1615/critreveukaryotgeneexpr.v14.i3.30

[9] Dyson N, Guida P, Munger K, Harlow E. Homologous sequences in adenovirus E1A and human papillomavirus E7 proteins mediate interaction with the same set of cellular proteins. J Virol. 1992;66(12):6893–6902. 1331501 10.1128/jvi.66.12.6893-6902.1992 PMC240306

[10] Jones DL, Alani RM, Munger K. The human papillomavirus E7 oncoprotein can uncouple cellular differentiation and proliferation in human keratinocytes by abrogating p21Cip1-mediated inhibition of cdk2. Genes Dev. 1997;11(16):2101–2111. 9284049 10.1101/gad.11.16.2101 PMC316455

[11] Gonzalez SL, Stremlau M, He X, Basile JR, Munger K. Degradation of the retinoblastoma tumor suppressor by the human papillomavirus type 16 E7 oncoprotein is important for functional inactivation and is separable from proteasomal degradation of E7. J Virol. 2001;75(16):7583–7591. 10.1128/JVI.75.16.7583-7591.2001 11462030 PMC114993

[12] Chandra J, Miao Y, Romoff N, Frazer IH. Epithelium Expressing the E7 Oncoprotein of HPV16 Attracts Immune-Modulatory Dendritic Cells to the Skin and Suppresses Their Antigen-Processing Capacity. PLoS One. 2016;11(3):e0152886 10.1371/journal.pone.0152886 27031095 PMC4816461

[13] Valenca JE, Goncalves AK, Guerreiro da Silva ID, Eleuterio Junior J, Tenorio da Silva T, Bruneska D, et al High Risk HPV E6/E7 Oncoprotein Expression in Women with High Grade Squamous Intraepithelial Lesion. Rev Bras Ginecol Obstet. 2016;38(3):154–159. 10.1055/s-0036-1580713 27022787 PMC10309481

[14] Wang N, Zhan T, Ke T, Huang X, Ke D, Wang Q, et al Increased expression of RRM2 by human papillomavirus E7 oncoprotein promotes angiogenesis in cervical cancer. Br J Cancer. 2014;110(4):1034–1044. 10.1038/bjc.2013.817 24423925 PMC3929894

[15] Chan PK. Human papillomavirus type 58: the unique role in cervical cancers in East Asia. Cell Biosci. 2012;2(1):17 10.1186/2045-3701-2-17 22571619 PMC3414832

[16] Gu Y, Ma C, Zou J, Zhu Y, Yang R, Xu Y, et al Prevalence characteristics of high-risk human papillomaviruses in women living in Shanghai with cervical precancerous lesions and cancer. Oncotarget. 2016. 10.18632/oncotarget.8262 PMC5029731 27013587

[17] Bao YP, Li N, Smith JS, Qiao YL, members A. Human papillomavirus type distribution in women from Asia: a meta-analysis. Int J Gynecol Cancer. 2008;18(1):71–79. 10.1111/j.1525-1438.2007.00959.x 17466054

