# Supplementary figures and images for: Production of Polyclonal Antibody to the HPV58 E7 Protein and Its Detection in Cervical Cancer
Source: PLoS One. 2016 Dec 29;11(12):e0169138. doi: 10.1371/journal.pone.0169138 (PMC5199089; doi:10.1371/journal.pone.0169138)

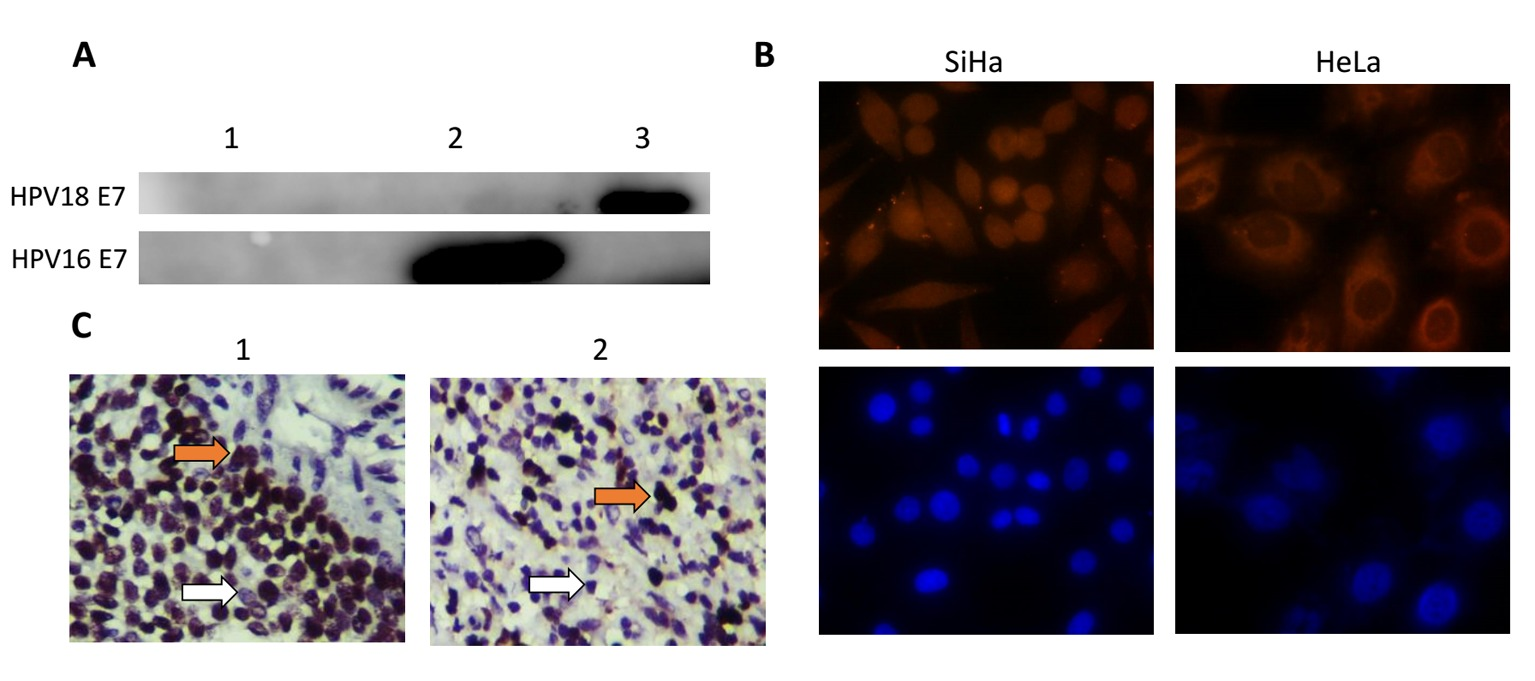

Supplement: S1 Fig — (A) western blotting for the detection of HPV16 and 18 E7. The upper band was marked with HPV18 E7 antibody. The lower band was marked with HPV16 E7. The Lanes 1, 2 and 3 are the pure protein of HPV 58, 16 and 18 E7. (B) Immunofluorescent analysis for the detection of HPV16 E7 in SiHa cells and HPV18 E7 in HeLa cells. (C) Immunohistochemistry stain for the detection of HPV16 and 18 E7 in cervical cancer cells. 1 is the HPV16-positive sample and 2 is the HPV18-positive sample. Red arrowheads indicate the positive cells. White arrowheads indicate the negative cells. (TIF) [file pone.0169138.s002.tif]
